# A role for caveola‐forming proteins caveolin‐1 and CAVIN1 in the pro‐invasive response of glioblastoma to osmotic and hydrostatic pressure

**DOI:** 10.1111/jcmm.15076

**Published:** 2020-02-17

**Authors:** Wenjun Pu, Jiawen Qiu, Zeyad D. Nassar, Paul N. Shaw, Kerrie‐Ann McMahon, Charles Ferguson, Robert G Parton, Gregory J. Riggins, Jonathan M. Harris, Marie‐Odile Parat

**Affiliations:** ^1^ School of Pharmacy The University of Queensland Brisbane Queensland Australia; ^2^ School of Medicine and Freemasons Foundation Centre for Men's Health South Australian Health and Medical Research Institute University of Adelaide Adelaide South Australia Australia; ^3^ Institute for Molecular Bioscience The University of Queensland Brisbane Queensland Australia; ^4^ Centre for Microscopy and Microanalysis The University of Queensland Brisbane Queensland Australia; ^5^ Department of Neurosurgery Johns Hopkins University School of Medicine Baltimore MD USA; ^6^ Institute of Health Biomedical Innovation Queensland University of Technology Brisbane Queensland Australia

**Keywords:** caveolae, extracellular matrix, invasiveness, mechanosensing, MMP‐2, MMP‐9, osmolality, tumour microenvironment, uPA

## Abstract

In solid tumours, elevated interstitial fluid pressure (osmotic and hydrostatic pressure) is a barrier to drug delivery and correlates with poor prognosis. Glioblastoma (GBM) further experience compressive force when growing within a space limited by the skull. Caveolae are proposed to play mechanosensing roles, and caveola‐forming proteins are overexpressed in GBM. We asked whether caveolae mediate the GBM response to osmotic pressure. We evaluated in vitro the influence of spontaneous or experimental down‐regulation of caveola‐forming proteins (caveolin‐1, CAVIN1) on the proteolytic profile and invasiveness of GBM cells in response to osmotic pressure. In response to osmotic pressure, GBM cell lines expressing caveola‐forming proteins up‐regulated plasminogen activator (uPA) and/or matrix metalloproteinases (MMPs), some EMT markers and increased their in vitro invasion potential. Down‐regulation of caveola‐forming proteins impaired this response and prevented hyperosmolarity‐induced mRNA expression of the water channel aquaporin 1. CRISPR ablation of caveola‐forming proteins further lowered expression of matrix proteases and EMT markers in response to hydrostatic pressure, as a model of mechanical force. GBM respond to pressure by increasing matrix‐degrading enzyme production, mesenchymal phenotype and invasion. Caveola‐forming proteins mediate, at least in part, the pro‐invasive response of GBM to pressure. This may represent a novel target in GBM treatment.

## 1 INTRODUCTION

Solid tumours are exposed to osmotic and mechanical stresses. Both preclinical and clinical studies indicate that tumours are generally poorly perfused, and tumour interstitial fluid pressure (IFP) is increased compared to normal tissue, with both increased hydrostatic pressure and oncotic pressure. This results from a combination of factors, including inefficient lymphatic drainage of soluble proteins, leaky and dysfunctional blood vessels, increased fibroblast numbers, thicker collagen fibres and accumulation of inflammatory factor‐secreting cells.^1^, ^2^, ^3^ Furthermore, in the case of solid tumours growing within the brain, the space limitation that the skull creates causes further compression as the tumour grows; patients with brain tumours experience increased intracranial pressure.^4^

Caveolae are flask‐shaped plasma membrane subdomains that require, for assembly, maintenance and functions, proteins from two families, the membrane‐embedded caveolins and the cytoplasmic cavins. Caveolae serve important roles in signalling, membrane homeostasis and mechanosensing.^5^ Caveolae also contribute to surface area homeostasis, as they flatten reversibly to allow instantaneous membrane tension buffering.^6^ The pressure‐sensing capabilities of caveolae and the mechanotransduction capacities of caveola‐forming proteins have pathophysiological consequences on cardiovascular or muscle function.^7^, ^8^ In glioblastoma (GBM), caveolae or caveola‐forming proteins have been proposed to be important in the regulation of EGFR signalling, resistance to treatment and exosome‐mediated cell‐cell communication.^9^, ^10^, ^11^ Moreover, the expression of both caveolin‐1 and CAVIN1 are increased in GBM compared to normal samples and are associated with shorter patient survival time and correlated with expression of the matrix proteases uPA and gelatinases.^12^

Matrix proteases are key mediators of the invasiveness of GBM, which in turn contributes to the disease particularly poor prognosis. Several signalling pathways known to be activated in GBM result in increased protease expression. Conversely, matrix proteases such as gelatinases or uPA act through both degradation of the brain extracellular matrix and the activation of pro‐migratory signalling.^13^ We have previously demonstrated that exposure of GBM adherent cell lines or oncospheres to osmotic and hydrostatic or centrifugal pressure induces an increase in GBM invasive potential in vitro. In the present study, we investigated whether caveolae mediate the pro‐invasive response to pressure in GBM cells.

## 2 MATERIALS AND METHODS

### 2.1 Materials

RPMI‐1640 medium, Opti‐MEM™ medium, geneticin, lipofectamine 3000, trypsin‐EDTA, penicillin/streptomycin, Coomassie brilliant blue R‐250 Pierce™ BCA Protein Assay Kit and real‐time PCR reagents were purchased from Life Technologies. The 40% acrylamide/bis solution was from Bio‐Rad. CultreCoat® 24‐well plates with BME‐Coated Inserts were from Bio Scientific Pty. Ltd. CultreCoat® 96 Well Medium BME Cell Invasion Assay Kit was from Bio Scientific Pty. Ltd. The caveolin‐1 primary antibody was from Cell Signaling Technology, and CAVIN1 primary antibody was from Proteintech®. ECL^TM^ Anti‐Rabbit IgG was purchased from GE Healthcare Science. Other reagents were purchased from Sigma‐Aldrich unless otherwise specified.

### 2.2 Cell culture

Human GBM cell line U118 was cultured in RPMI medium supplemented with 10% (v/v) FBS, 100 U/mL penicillin and 100 μg/mL streptomycin. U251 cells were transfected with plasmids encoding shRNA to CAV1 or control shRNA using Transpass™ according to the manufacturer's instructions. Stably transfected clones were isolated after selection using 250 μg/mL G418 and tested using Western blot analysis with anti‐CAV1 and anti‐CAVIN1 primary antibodies. All cell lines were incubated at 37°C with 5% CO_2_.

### 2.3 Generation of CAVIN1 or caveolin‐1 CRISPR knockout U251 cell lines

CAV1 or CAVIN1 clustered regularly interspaced short palindromic repeats (CRISPR) knockout U251 cell lines were generated at the Queensland Facility for Advanced Genome Editing (QFAGE), Institute for Molecular Bioscience, The University of Queensland. For each gene, three guide RNA (gRNA) targeting the coding sequence of exon1 or exon2 was designed using the free online tool http://crispr.mit.edu/. For CAVIN1, the sequences were gRNA1: ATCAAGTCGGACCAGGTGAA, gRNA2: GCTCACCGTATTGCTCGTGG, gRNA3: GTCAACGTGAAGACCGTGCG; for caveolin‐1, the sequences were gRNA1: ATGTTGCCCTGTTCCCGGAT, gRNA2: AGTGTACGACGCGCACACCA, gRNA3: GTTTAGGGTCGCGGTTGACC. Ribonucleoprotein (RNP) complexes were formed by mixing 20 pmol of each synthesized gRNA (crRNA + tracrRNA, IDT) with 20 pmol of spCas9 protein. Assembled RNPs were combined with 200 000 U251 cells and transfected using a Lonza Nucleofector 4D device (kit SE, program DS‐138). Forty‐eight hours post‐transfection, genomic DNA of bulk transfected cell pools was extracted and the editing efficacy was confirmed by T7E1 (T7 Endonuclease I) assay and Sanger sequencing analysis. Pooled cells transfected with the three gRNA were tested for loss of CAV1 and CAVIN1 protein expression.

### 2.4 siRNA‐mediated CAV1 or CAVIN1 knockdown

CAV1 or CAVIN1 expression was inhibited in U251 cells using Stealth siRNAs. The CAV1 Stealth siRNAs (HSS141466, HSS141467 and HSS141468), CAVIN1 Stealth siRNAs (HSS138488, HSS138489 and HSS178652) and Stealth RNAi™ siRNA Negative Control, Med GC were purchased from Life Technologies (Life Technologies). The transfection was conducted using Lipofectamine 3000 as previous described. After the transfection, the cells were split as required for experiments.

### 2.5 Osmotic stress

The different osmolality media were prepared as previously described 12 and controlled using an OSMOMAT 3000 basic freezing point osmometer (Gallay) calibrated using 300 and 500 mOsmol/kg standards. The osmolalities employed to study cellular responses were selected based on cell survival at 48 hours For U118, U87, U251 and shRNA U251 cells, the highest non‐toxic osmolality was 440 mOsmol/kg. For CRISPR‐Cas9 cells, the highest hyperosmolality was 360 mOsmol/kg. 1.0 × 10^6^ cells were seeded in 12‐well plates and incubated at 37°C with 5% CO_2_ for 24 hours After 24 hours, cells were rinsed with serum‐free medium twice and 1 mL of serum‐free medium of varying osmolality was added to each well and incubated for 48 hours The medium was collected and centrifuged at 935 *g* for 5 minutes then stored at −80°C until analysis.

### 2.6 Hydrostatic pressure treatment

5.0 × 10^6^ cells were seeded in T25 flask and incubated at 37°C with 5% CO_2_ for 24 hours Cells were rinsed with serum‐free medium twice and added with 3 mL of serum‐free medium containing 25 µmol/L HEPES. The flask lid was fitted with a three‐way stopcock (BD Connecta™), and pressure increased to 30 mm Hg using a sphygmomanometer. The pressure was maintained by closing the stopcock, and the cells were incubated for 48 hours A flask with an air‐permeable lid was used as control. The medium was collected and centrifuged at 935 *g* for 5 minutes then stored at −80°C until analysis.

### 2.7 MTT assay

The cell viability was tested using the 3‐[4,5‐dimethylthiazole‐2‐yl]‐2,5‐diphenyltetrazolium bromide (MTT) assay as previous described. The UV absorbance was read with an iMark™ microplate absorbance reader at 595 nm (Bio‐Rad Laboratories). Background absorbance was subtracted, and results were expressed as the percentage viability of control cells.

### 2.8 In gel zymography

Gelatinases and uPA in media conditioned by different cell lines were measured by gelatin or casein‐plasminogen zymography as previous described. The gels were scanned, and uPA, MMP‐2 and MMP‐9 were quantified by densitometry using Image J (v1.48) software.

### 2.9 Quantitative RT‐PCR

The mRNA expression of specific genes and epithelial‐to‐mesenchymal transition (EMT) markers levels was measured by real‐time reverse transcriptase‐polymerase chain reaction (real‐time RT‐PCR). Total RNA was isolated and purified using the PureLink® RNA Mini Kit (Life Technologies). The total RNA (2000 ng) was reverse transcribed using the High‐Capacity cDNA Reverse Transcription Kit (Life Technologies). Quantitative real‐time PCR was performed using TaqMan™ Fast Universal PCR Master Mix (Life Technologies) in a StepOnePlus 7500 real‐time PCR system (Applied Biosystems). The primers of target genes used for this analysis were TaqMan™ Gene Expression Assay for human PLAU (Hs01547054_m1), PLAUR (Hs00958880_m1), MMP‐2 (Hs01548727_m1), MMP‐9 (Hs00957562_m1), CAV1 (Hs00971716_m1), CAVIN1 (Hs00396859_m1), AQP1 (Hs01028916_m1), Snail‐1 (Hs00195591_m1), Snail‐2 (Hs00161904_m1), Twist (Hs01675818_s1), Vimentin (Hs00185584_m1) and N‐cadherin (Hs00983056_m1). Relative quantification was done by reference to 18S ribosomal RNA (18S rRNA) and analysed using the comparative critical threshold (Ct) method.^14^

### 2.10 Electron microscopy

U251 cells were processed for transmission electron microscopy in 3 cm dishes using standard protocols.^15^ Images were taken at a magnification of 12 000×. The number of surface clathrin‐coated pits (CCP), surface caveolae (where a clear connection to the plasma membrane was evident) and putative caveolae (vesicular profiles < 100 nm close to the plasma membrane but with no clear connection to the plasma membrane) per cell profile was counted in 12 cell profiles per condition, from two different areas of the culture dish.

### 2.11 Western blotting assay

Equal amounts of protein from cell lysates were electrophoresed in an 11% SDS‐PAGE gel and transferred to a nitrocellulose membrane. The proteins of interest were identified using rabbit anti‐caveolin‐1 polyclonal antibody (1:1000) or rabbit anti‐CAVIN1 polyclonal antibody (1:1000) followed by secondary antibody (1:10 000), detected using SuperSignal™ West Dura Extended Duration Substrate (Life Technologies) and quantified using a ChemiDoc^TM^ Touch Imaging System (Bio‐Rad).

### 2.12 Cell invasion assay

The cell invasion assay was performed using either CultreCoat® 24‐well plates with BME‐Coated Inserts or CultreCoat® 96 Well Medium BME Cell Invasion assay as previously described^12^; the upper and bottom chambers had the same concentration of NaCl added.

### 2.13 mRNA expression and survival analysis from publicly available data

AQP‐1 mRNA expression in normal brain tissues and different GBM molecular subtypes was retrieved from Project Betastasis web platform (http://www.betastasis.com). Data were extracted from The Cancer Genome Atlas (TCGA) consortium using Affymetrix Human Exon 1.0 ST platform. The four molecular subtypes of GBM (classical, mesenchymal, neural or proneural) were defined based on gene expression profiles 16 and have significance for survival outcome or therapy response. For the survival analysis, AQP‐1 mRNA expression using U133 microarray and survival data was extracted from glioblastoma multiforme (TCGA, Firehorse Legacy), accessed through CBioPortal.^17^, ^18^ The Kaplan–Meier curves, log‐rank test and Cox multivariable regression analysis were generated using GraphPad Prism (version 8.01).

### 2.14 Statistical analysis

Statistical analysis was carried out using GraphPad Prism software (v. 8.01). *P*‐value of <.05 was considered significant. Data show either replicates or independent experiments as detailed in the figure legends. All the data are shown as mean ± SEM.

## 3 RESULTS

### 3.1 Osmotic pressure increases caveola‐forming protein expression

**Figure 1:**
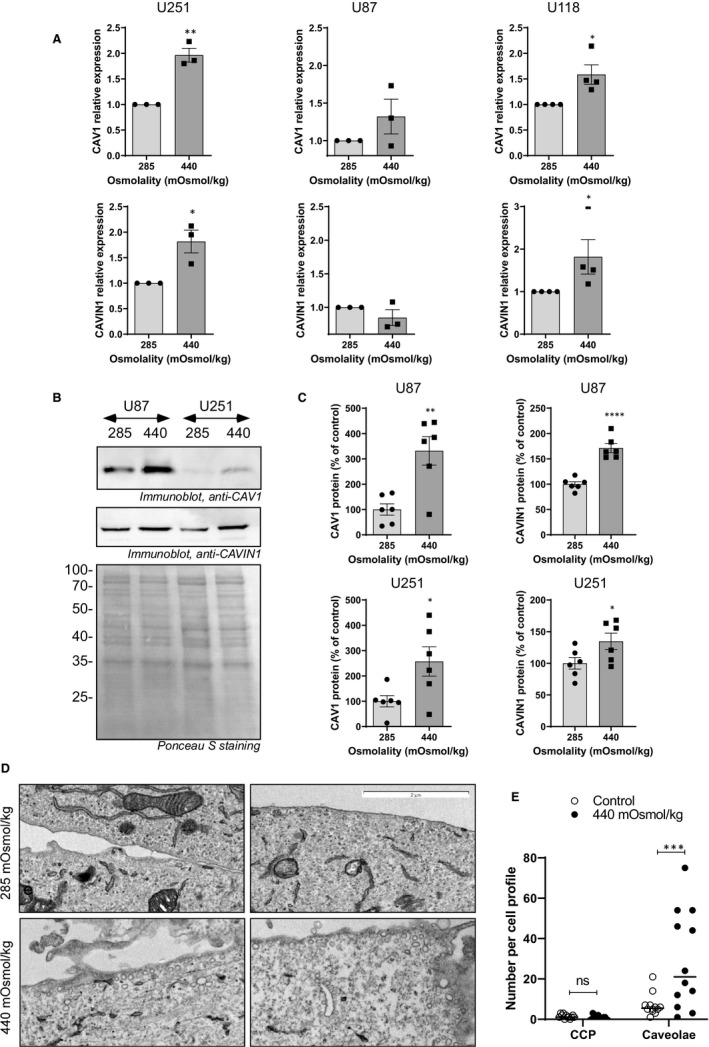
Osmotic pressure increases caveola‐forming protein expression. Cells were subjected to normo‐ (285 mOsmol/kg) or hyper (440 mOsmol/kg)‐osmolar medium. (A) Effect of osmotic pressure on CAV1 and CAVIN1 mRNA expression among U251, U87 and U118 cell lines. Results are expressed as mean ± SEM of n = 3 independent experiments and analysed using unpaired one‐tailed t test. (B) Effect of osmotic pressure on CAV1 and CAVIN1 protein expression in U87 and U251 cell lines detected by immunoblotting. (C) Results of densitometric quantitation are shown as a % of control osmolality. Results are expressed as mean ± SEM of n = 6 determinations and analysed using unpaired one‐tailed t test. (D) Representative electron micrographs of U251 cells exposed to iso‐ (285 mOsmol/kg) or hyper (440 mOsmol/kg)‐osmolar medium for 48 h (E) quantification of clathrin‐coated pits (CCP) and caveolae in 12 cell profiles per condition. Results were analysed by one‐way ANOVA with Dunnett's multiple comparisons test or two‐way ANOVA with Sidak's multiple comparison test. *P < .05, **P < .01, *** *P* < .001, ****P < .0001

We have previously demonstrated that the expression of caveolin‐1 and CAVIN1 correlates with GBM invasiveness 12 and that GBM cells respond to osmotic pressure by increased production of matrix‐degrading enzymes, EMT markers and invasion.^19^ We asked whether caveolae may be involved in the cellular response to pressure. We tested the mRNA expression of CAV1 and CAVIN1 in GBM cells exposed to hyperosmotic or hypo‐osmotic medium for 48 hours (Figure 1A). The osmolality was adjusted by adding sodium chloride as previously described.^19^ Hyperosmolality (440 mOsmol/kg) caused a slight increase (around 2‐fold) in mRNA of CAV1 and CAVIN1 in the U251 cell line. A similar trend was seen in the U87 and U118 cell lines for CAV1 mRNA, albeit non‐statistically significant, and for CAVIN1 in U118 cells. Of note, the U118 cell line spontaneously lacks expression of these two proteins essential for caveola formation.^12^ CAV1 and CAVIN1 protein expression was tested after incubation of the U87 and U251 cell lines in control and hyperosmolar media (Figure 1B), and a statistically significant increase of both proteins was seen in both cell lines (Figure 1C). Caveola quantification was performed on electron micrographs of U251 cells exposed to control or hyperosmolar medium for 48 hours (Figure 1D). Hyperosmolality resulted in a significant increase in the number of caveolae, whereas the number of clathrin‐coated pits was unaltered.

### 3.2 Caveolae are required for GBM pro‐invasive response to hyperosmotic pressure

**Figure 2:**
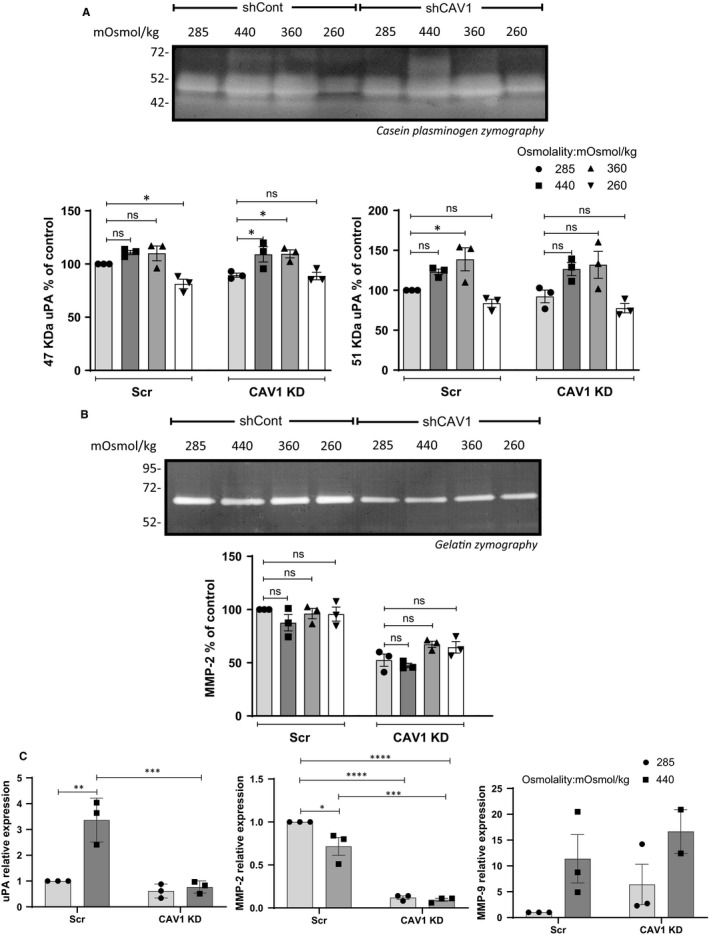
Effect of osmotic pressure on matrix protease production of U251 cells stably expressing shRNA to CAV1 (shCAV1) or control shRNA (shCont). Cells were subjected to normo‐ (285 mOsmol/kg), hyper‐ (360 or 440 mOsmol/kg) or hypo (260 mOsmol/kg)‐osmolar medium. (A) Conditioned media of U251 shCont and shCAV1 cells exposed to osmotic stress were analysed by casein‐plasminogen zymography and densitometric quantitation of the 47 and 51 kD bands corresponding to uPA was carried out. (B) Gelatin zymography and densitometric quantitation of MMP‐2 produced in the conditioned medium of U251 shCont and shCAV1 cells after 48 h of osmotic stress. (C) Effect of osmotic stress on uPA, MMP‐2 and MMP‐9 mRNA expression. Results are shown relative to shCont. All results are expressed as mean ± SEM of n = 3 independent experiments. Densitometric quantification of uPA and MMP‐2 was analysed by one‐way ANOVA with Dunnett's multiple comparisons test; protease mRNA expression was analysed by two‐way ANOVA with Tukey's multiple comparisons test. *P < .05, **P < .01, ***P < .001, ****P < .0001

**Figure 3:**
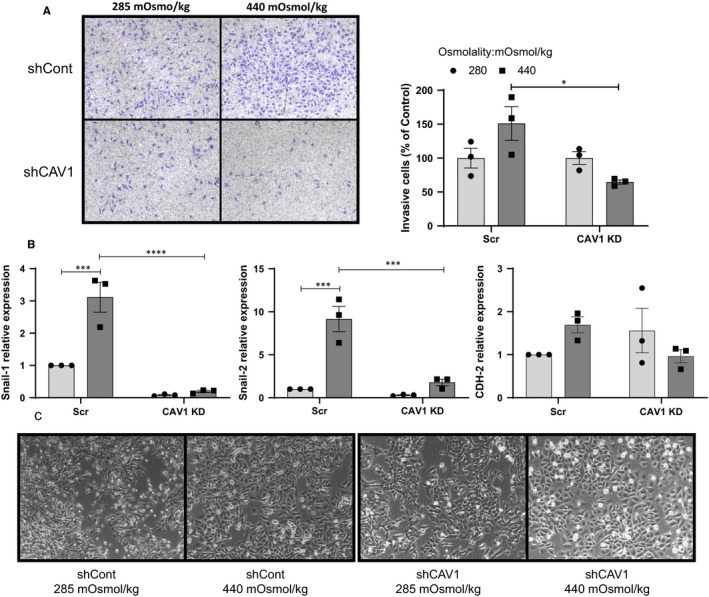
Effect of osmotic pressure on U251 shCont and shCAV1 cells in vitro invasion potential. Cells were subjected to normo‐ (285 mOsmol/kg) or hyper (440 mOsmol/kg)‐osmolar medium. (A) Cell invasion through Matrigel‐coated Transwells was determined in 285 or 440 mOSmol/kg media. Quantitation of invaded cells is shown as per cent of normo‐osmotic control. Representative micrographs are shown. (B) mRNA expression of EMT markers in U251 shCont and shCAV1 cells after 48 h of osmotic stress. (C) Morphology of U251 shCont and shCAV1 cells after 48 h in 285 or 440 mOSmol/kg media. Results are expressed as mean ± SEM of n = 3 independent experiments, *P < .05, ***P < .001, ****P < .0001, two‐way ANOVA analysis with Tukey's multiple comparisons test

We assessed whether caveolae are required for the pro‐invasive response to pressure by quantifying uPA, gelatinase and EMT marker expression in U251 cells in which CAV1 expression was stably down‐regulated by shRNA.^12^ The cells were subjected to hyperosmolarity (360 and 440 mOSmol/kg) or hypo‐osmolarity (260 mOsmol/kg) and compared to cells placed in normo‐osmolar (285 mOsmol/kg) medium. CAV1 down‐regulation did not affect the increased production of uPA in response to 360 or 440 mOsmol/kg (Figure 2A) as was apparent from casein‐plasminogen zymography results. Furthermore, no increase in MMP‐2 was seen, and MMP‐9 was undetected by gelatin zymography in either control (scramble shRNA) or CAV1 down‐regulated cells (Figure 2B). However, uPA mRNA was significantly increased in 440 mOsmol/kg medium (Figure 2C) and this response was blunted in CAV1 knocked down cells. MMP‐2 mRNA was not increased in response to pressure, and MMP‐9 mRNA was increased to the same extent in control and CAV1 knocked down cells (ie 10‐15 fold, no statistically significant difference; Figure 2C). Control (scrambled shRNA‐transfected) cells invaded ~ 50% more when placed in hyperosmotic medium compared to normo‐osmotic medium, a response which was dampened in CAV1 down‐regulated cells (Figure 3A). When analysed for mRNA expression of EMT markers, cells with down‐regulated CAV1 expression expressed significantly less Snail‐1 and Snail‐2 in response to osmotic stress compared to the scrambled shRNA control cells (Figure 3B). The morphology of the cells after 48 hours in control or hyperosmolar medium was similar in both cell lines, with an already mesenchymal, elongated appearance in control medium and increased apparent cell spreading and coverage of the plastic surface in 440 mOsmol/kg media (Figure 3C). These results indicate that caveolae may mediate some of the invasive features induced by osmotic stress in GBM cells.

To confirm these findings, we assessed the response to osmotic pressure of U118 cells subjected to hyperosmotic or hypo‐osmotic medium. The osmolalities employed were verified not to decrease cell viability (Figure S1a). The caveola‐devoid U118 cells failed to increase production of uPA, MMP‐2 and/or MMP‐9 in conditioned medium (Figure S1b,c). Similarly, in the absence of caveolae, the induction of Snail‐2/Slug or cadherin2 (CDH2) mRNA by hyperosmolar medium (440 mOsmol/kg) was lost in U118 cells. The increase in MMP‐9 mRNA and Snail‐1 mRNA, however, was still apparent with 440 mOsmol/kg (Figure S1d,e). We also tested invasion through Matrigel‐coated inserts. U118 cells did not invade through the Matrigel layer whether in the presence of control (258 mOsmol/kg) or hyperosmotic (440 mOsmol/kg) medium (data not shown). Of note, the lack of caveola‐forming protein expression is not the only difference between U118 cells and U87 or U251 cells; in unstimulated experimental conditions, U118 also exhibit lower mRNA expression of the EMT markers CDH2, Twist and vimentin compared to U87 or U251 (Figure S1f).

**Figure 4:**
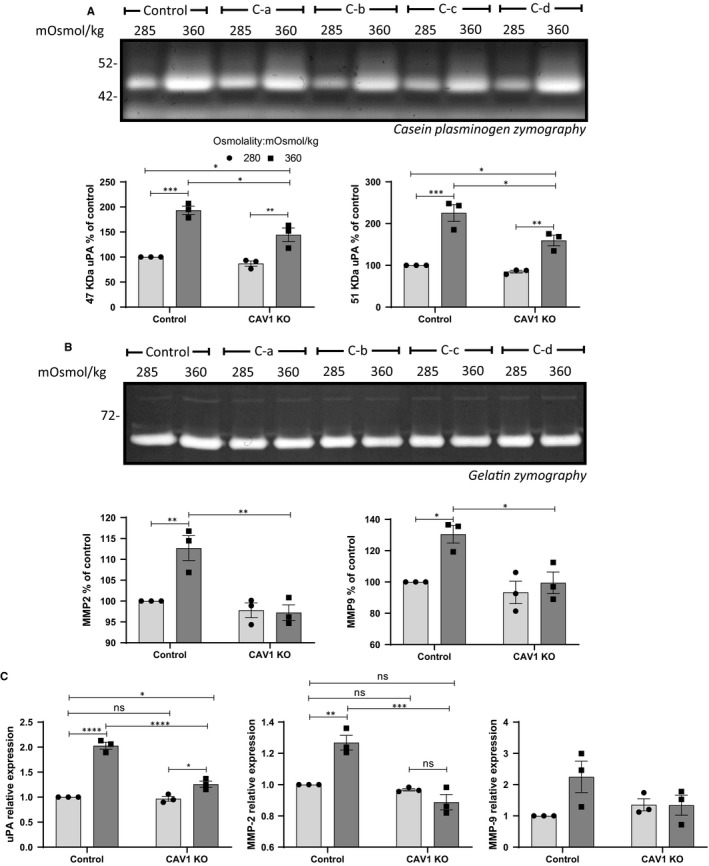
Effect of osmotic pressure on matrix protease production of CAV1 CRISPR‐Cas9 knockout (KO) U251 cells. Cells were subjected to normo‐ (285 mOsmol/kg) or hyper (360 mOsmol/kg)‐osmolar medium. (A) Conditioned media of control and CAV1 KO cells exposed to osmotic stress were analysed by casein‐plasminogen zymography. Densitometric quantitation of the 47 and 51 kD bands corresponding to uPA was carried out. (B) Gelatin zymography and densitometric quantitation of MMP‐2 and MMP‐9 produced in the conditioned medium of control and CAV1 KO cells after 48 h of osmotic stress. (C) Effect of osmotic stress on uPA, MMP‐2 and MMP‐9 mRNA expression. The results are the average of n = 4 KO cell pools. Results are expressed as mean ± SEM of n = 3 independent experiments, *P < .05, **P < .01, ***P < .001, ****P < .0001, two‐way ANOVA analysis with Tukey's multiple comparisons test

We further tested U251 cells in which the down‐regulation of CAV1 or CAVIN1 was successfully achieved using siRNA oligos as previously described.^12^ The results indicate that the down‐regulation of either CAV1 (Figure S2a,b) or CAVIN1 (Figure S2c,d) did not affect the cell response to pressure when we quantified the mRNA expression of matrix proteases (Figure S2a,c) or EMT markers (Figure S2b,d). These results indicated that the effect of down‐regulation of caveola‐forming proteins may take time to appear and suggested the use of stable and complete ablation of CAV1 or CAVIN1 using CRISPR knockout GBM cells. We compared the response to hyperosmolality of U251 cells in which CAV1 expression was depleted using three separate guides (pools C‐a, C‐b, C‐c) or the three guides together (C‐d). While in the absence of CAV1 cells still increased their production of uPA as measured by casein‐plasminogen zymography, the amount of uPA was significantly less in the CAV1 KO cells compared to the control cells (Figure 4A). The quantitative results for each individual pool of KO cells are presented in Figure S3a. Although the increase in MMP‐2 in response to pressure was much less dramatic than the increase in uPA, comparable results were found when analysing the gelatin zymographs (Figure 4B, Figure S3a). At the mRNA level, the induction of uPA, MMP‐2 and MMP‐9 mRNA by hyperosmolar medium was blunted by CAV1 KO, although statistically significant only for uPA and MMP‐2 (Figure 4C, Figure S3b).

**Figure 5:**
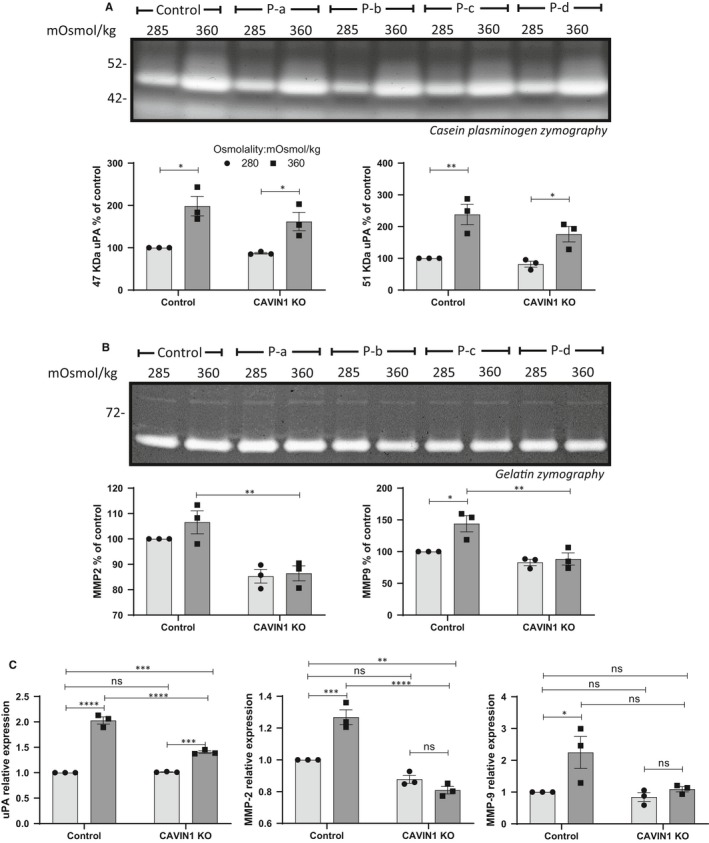
Effect of osmotic pressure on matrix protease production of CAVIN1 CRISPR‐Cas9 KO U251 cells. Cells were subjected to normo‐ (285 mOsmol/kg) or hyper (360 mOsmol/kg)‐osmolar medium. (A) Conditioned media of control and CAVIN1 KO cells exposed to osmotic stress were analysed by casein‐plasminogen zymography. Densitometric quantitation of the 47 and 51 kD bands corresponding to uPA was carried out. (B) Gelatin zymography and densitometric quantitation of MMP‐2 and MMP‐9 produced in the conditioned medium of control and CAVIN1 KO cells after 48 h of osmotic stress. (C) Effect of osmotic stress on uPA, MMP‐2 and MMP‐9 mRNA expression. The results are average of n = 4 KO pools. Results are expressed as mean ± SEM of n = 3 independent experiments, *P < .05, **P < .01, ***P < .001, ****P < .0001, two‐way ANOVA analysis with Tukey's multiple comparisons test

Similar data were obtained when analysing the response to hyperosmolality in U251 cells in which CAVIN1 was ablated using CRISPR knockout (Figure 5); uPA was increased in the CAVIN1 KO cells in response to hyperosmolality but this response was lower than that seen in control cells (Figure 5A, Figure S4a). The increase in MMP‐9 production was also blunted in the CAVIN1 KO cells (Figure 5B, Figure S4a). At the mRNA level, the induction of uPA, MMP‐2 and MMP‐9 by hyperosmolality was decreased or ablated in the CAVIN1 KO cells (Figure 5C).

**Figure 6:**
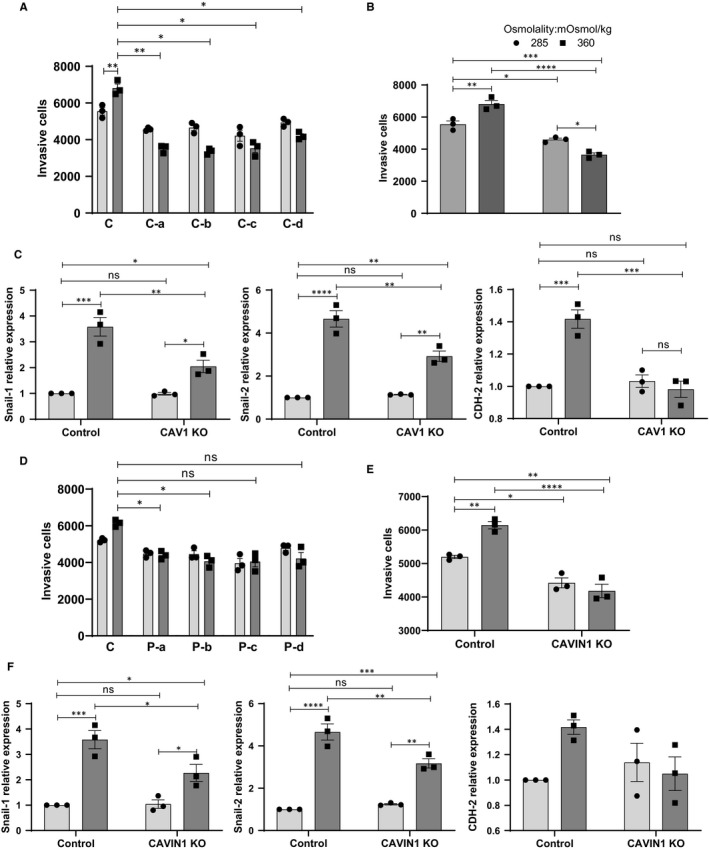
Effect of osmotic pressure on CAV1 or CAVIN1 CRISPR‐Cas9 KO U251 cells in vitro invasion potential. (A) Determination of basement membrane cell invasion of control or four individual CAV1 KO pool in 285 or 360 mOSmol/kg media. (B) Quantification of CAV1 KO cell invasion shown as the average of n = 4 independent CAV1 KO cell pools. (C) mRNA expression of EMT markers in CAV1 KO cells after 48 h of osmotic stress. (D) Determination of basement membrane cell invasion of control or four individual CAVIN1 KO cell pools in 285 or 360 mOSmol/kg media. (E) Quantification of CAVIN1 KO cell invasion shown as the average of n = 4 independent CAVIN1 KO cell pools. (F) mRNA expression of EMT markers in CAVIN1 KO cells after 48 h of osmotic stress. All results are expressed as mean ± SEM of n = 3 independent experiments. Results were analysed by one‐way ANOVA with Dunnett's multiple comparisons test (A, D) or two‐way ANOVA with Tukey's multiple comparisons test (B, C, E, F). *P < .05, **P < .01, ***P < .001, ****P < .0001

Lastly, we examined the response of the caveolin and cavin KO cells to exposure to hyperosmolar medium (360 mOsmol/kg rather than 440 mOsmol/kg to maintain cell viability; see Figure S5) for 24 or 48 hours In contrast to WT cells, the KO cells did not show a significant response to the high osmolality medium in invasion assays (Figure 6A,B,D,E). Increased expression of EMT markers in response to pressure was also significantly prevented by knockout of CAV1 (Figure 6C) or CAVIN1 (Figure 6F).

### 3.3 Caveolae are required for GBM pro‐invasive response to hydrostatic pressure

Previous work has shown that GBM respond to hydrostatic pressure by increasing matrix proteases and EMT marker expression,^19^ and caveolae have been proposed to serve mechanosensing roles. Therefore, in the following set of experiments, we assessed whether CRISPR ablation of caveola‐forming proteins CAV1 or CAVIN1 altered the pro‐invasive response of U251 cells to hydrostatic pressure, that is 30 mm Hg applied to the cells over 48 hours, as previously described.^19^ The mRNA expression of matrix proteases (Figure S6a,c) and EMT markers (Figure S6b,d) in response to increased hydrostatic pressure was significantly lower in cells lacking caveola‐forming proteins CAV1 or CAVIN1 for the majority of the genes assessed.

### 3.4 Hyperosmolarity‐induced aquaporin‐1 mRNA expression is lost upon caveola ablation

**Figure 7:**
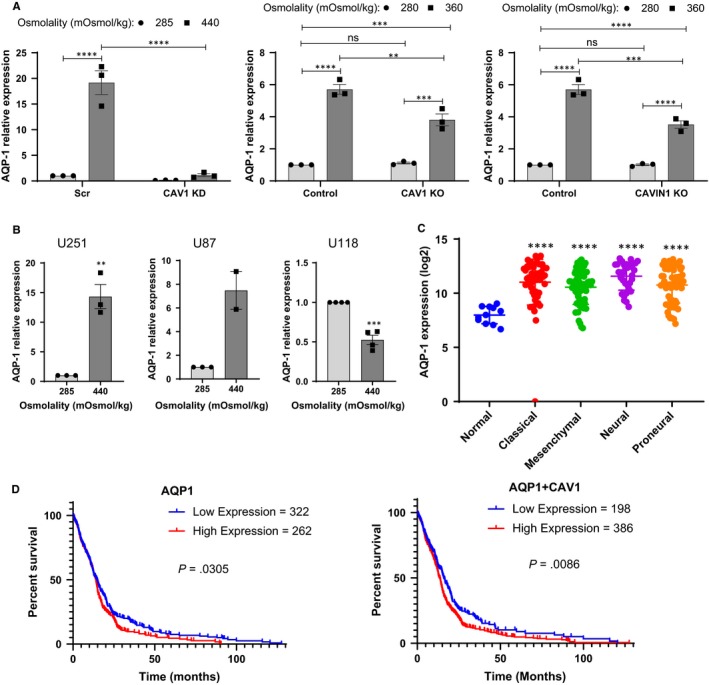
Interplay between caveolae, osmotic pressure and aquaporin 1 (AQP‐1) expression in GBM cell lines. Cells were subjected to normo (285 mOsmol/kg)‐ or hyper (360 or 440 mOsmol/kg)‐osmolar medium. (A) Effect of osmotic pressure on AQP‐1 mRNA expression among CAV1 shRNA knocked down, CAV1 CRISPR‐Cas9 KO and CAVIN1 CRISPR‐Cas9 KO U251 cells. (B) Effect of osmotic pressure on AQP‐1 mRNA expression among U251, U87 and U118 cell lines. (C) Dots plot for AQP‐1 expression (log base 2 transformed data) in normal brain tissues and in different GBM molecular subtypes in TCGA dataset (normal, n = 11; classical, n = 54; mesenchymal, n = 58; neural, n = 33; proneural, n = 57). (D) Kaplan‐Meier plots of GBM patient survival based on AQP‐1 or AQP1 and CAV1 expression. Survival probability is shown as a function of time after diagnosis (months) classified by mRNA expression. Expression (*z* > .5) was chosen as cut‐off between “high” and “low” expression

Aquaporins are membrane water channels necessary for cellular response to osmotic stress. AQP1 and AQP4 are highly expressed in GBM, but only AQP1 is expressed by the three cell lines U87, U118 and U251. We determined whether GBM cells exposed to hyperosmolality increased aquaporin‐1 (AQP1) mRNA expression in response to hyperosmolar stress. We tested U251 cells in which CAV1 expression was knocked down *via* shRNA, and U251 cells KO for CAV1 or CAVIN1 using CRISPR (Figure 7A). In all three loss‐of‐function experiments, the loss of caveola‐forming proteins significantly impaired the ability of the cells to increase APQ1 mRNA in response to hyperosmolality. We further tested cell lines expressing or spontaneously lacking caveola‐forming proteins (Figure 7B). In U251 and U87 cells, hyperosmolality increased AQP1 mRNA expression. Interestingly, in caveola‐forming protein‐deficient U118 cells, hyperosmolality did not result in increased AQP1 mRNA induction (Figure 7B). Lastly, we interrogated publicly available data to determine the significance of AQP1 expression in GBM. Compared to normal brain tissue, AQP1 was overexpressed in all molecular subtypes (Figure 7C). Furthermore, these analyses indicated that survival of patients with tumours expressing high APQ1 levels was shorter when compared to patients with low AQP1 expression and that the difference in survival was more significant in the case of joint high expression of AQP1 and CAV1 (Figure 7D).

## 4 DISCUSSION

Our results show that caveolae mediate, at least in part, the pro‐invasive response of GBM to osmotic and hydrostatic pressure. It has been shown irrefutably that the absence of the key caveola‐forming proteins CAV1 or CAVIN1 prevents the formation of caveolae.^20^, ^21^ Caveolae protect cells from mechanical stress *via* a combination of mechanisms; they provide a membrane reservoir which can be deployed when caveolae disassemble, flatten and buffer tensions occurring, for example, in myofibre lengthening,^7^ and in response to hypo‐osmotic stress,^7^, ^22^ haemodynamic forces 23 or tether pulling.^6^ It is also proposed that caveolae mediate the internalization of damaged membrane areas.^24^ Lastly, the disassembly and flattening of caveolae in response to membrane stretch allows the release of cavins to signal intracellularly (including in the nucleus).^6^, ^7^, ^25^

In the present study, we did not observe major effects of hypo‐osmotic treatment on matrix protease production or EMT marker expression. Hypo‐osmotic stress has been used at various intensities for limited amounts of time in other documented experiments testing the role of caveolae in mechano‐protection.^7^, ^22^ These experiments unveiled a physiological role for caveolae in limiting membrane tension in response to cell stretch or swelling 22 and an increased susceptibility to hypo‐osmotic stress and impaired membrane integrity in CAVIN1^‐/‐^ muscle fibres.^7^ Hypo‐osmotic medium was made by diluting DMEM 10‐fold with 10% FBS‐containing water,^23^ applying hypo‐osmotic medium at 30 mOsm instead of 300mOsm 6 and applying a HEPES‐based solution at 180 mOsm vs isosmotic 280 mOsm.^22^ Durations were 10‐15 minutes.^6^, ^7^, ^22^, ^23^ In the present study, hypo‐osmotic medium was applied for a much longer duration (48 hours) and at an intensity compatible with cell survival (tested by MTT). This is a major difference with existing published work where rapid cell swelling was employed.

The literature linking hyperosmotic stress to caveolae is limited. Early work indicated that hyperosmotic stress caused Src family kinase‐dependent caveolin‐1 tyrosine phosphorylation after short‐term (10‐15 minutes) exposure to mannitol.^26^, ^27^ Furthermore, 30‐min hyperosmotic stress using sorbitol was reported to induce internalization of CAV1.^28^ More recently, plasma membrane repair was shown to be increased in hyperosmotic medium (using a 10‐min incubation in 340 mOsmol/kg medium) and, interestingly, this hyperosmotic stress caused a transient down‐regulation of clathrin and fluid phase endocytosis while stimulating caveolar endocytosis.^29^ In our experiments, GBM cells were exposed to long duration (48 hours) hyperosmolality, being achieved by NaCl addition to the medium. It is reasonable to speculate that, over this extended period of time, the cells adapted to environmental pressures to maintain volume homeostasis. Cell shrinking is exploited by GBM cells to invade their surroundings 30 and is mediated at least in part by volume‐regulated anion channels, normally involved in re‐establishing cell volume after a swelling event (*eg* exposure to hypotonic medium or hypoxia).^31^ In contrast, when exposed to hypertonic medium, GBM cells have been shown to shrink within 2‐3 minutes and then to regulate their volume back to normal within 25 minutes, a response which involves Na^+^‐K^+^‐2Cl^−^ co‐transporter isoform 1.^32^ Our results show that U251 cells respond to long‐term (48 hours) hypertonic exposure by increasing the expression of caveola‐forming proteins CAV1 and CAVIN1, and the water‐transporting protein AQP1. GBM tissues show increased AQP1 expression, and patients with high AQP1 had significantly lower survival time. While it is tempting to hypothesize that AQP1 contributes to the aggressiveness of these tumours, it is possible that high expression of APQ1 is a consequence of the higher pressure that exists inside the tumour. Future work is required to investigate whether pharmacological or molecular ablation of AQPs modulates pressure‐induced GBM invasion. Furthermore, in the absence of caveolae, induction of AQP1 mRNA expression and GBM pro‐invasive response to hyperosmolality were both dampened, indicating an interplay between caveolae and AQP1 in pressure‐induced invasion that is yet to be explored. Future experiments will assess the impact of knocking down AQP1 on hyperosmolarity‐driven invasiveness. In favour of a clinically significant interaction, the effect of high expression of CAV1 and AQP1 on survival of GBM patients was more significant than that of high expression of AQP1 alone. AQP1 localization in caveolae is unclear from the literature; in endothelial cells from rat lung, around 70% of AQP1 was located in caveolae with the remainder located in non‐caveolar plasma membrane.^33^ Similar results have been found in mouse 34 and in human heart.^35^ In rat cardiac myocytes, the AQP1 co‐localization with caveolin‐3 could be reversed by hyperosmotic medium.^36^ On the contrary, AQP1 was shown to have a low level of co‐localization with caveolae in rat lung 37 and brain microvascular endothelial cells.^38^ These differences may be due to cell‐type specificity or dissimilarities in osmotic conditions. In addition to co‐localization, a functional link between aquaporins and caveolae has been explored. In a bladder outlet obstruction rat model, both AQP1 and CAV1 expression increased with high contraction pressure 39 and in CAV1 gene‐disrupted mice, an increased expression of AQP1 was associated with bladder dysfunction.^40^ In addition, in an animal study, down‐regulation of CAV1 *via* siRNA decreased the expression of AQP1 and AQP5 with increasing lung oedema.^41^ Taken together, a complex picture emerges regarding the relation between AQP1 and caveola‐forming proteins. We interrogated two TCGA data sets but found no positive correlation between caveola‐forming protein and AQP1 mRNA expression in GBM (data not shown).

Our results further indicate the involvement of caveolae in the response of GBM to hydrostatic pressure. It has been proposed that the IFP is uniformly high in the centre of a solid tumour and decreases abruptly at the periphery.^42^ Furthermore, high IFP in tumours correlates with metastasis in vivo*;* high central tumour IFP was found to be associated with the development of pulmonary and lymph node metastases in a model of human melanoma xenograft.^43^ High tumour IFP in cervical cancer patients was also found to significantly predict local or distant recurrence of the tumour.^44^ The pressure model employed (by forcing air into a flask and leaving the pressure on for 48 hours) results in continuous, long‐term pressure. The pressure that we employed (30 mm Hg) is compatible with what has been measured in clinical and preclinical studies 43, 44 and was also used in in vitro experiments.^45^ In a study of lung cancer cells exposed to 20 mm Hg,^45^ increased migration was shown starting 4‐6 hours after applying pressure; the cells became thinner but spread wider, proliferated more, produced more filopodia, invaded more and expressed higher amounts of Snail—all of these corroborate our finding that 30 mm Hg increases GBM invasive potential at 48 hours Furthermore in this study of lung cancer cells, the expression of AQP1 increased with hydrostatic pressure and AQP1 knock down dampened the motile response of these cells to pressure.^45^ Our results did not confirm this increased expression in AQP1 in response to hydrostatic pressure, presumably because of the difference in time‐points used in each study (8 hours vs 48 hours) and the end‐point measurement (mRNA expression vs protein). Interestingly, this previous study indicated that AQP1 was downstream of CAV1 because CAV1 SiRNA KD prevented pressure‐induced AQP1 elevation,^45^ which matches our findings with osmotic but not hydrostatic pressure.

Overall, our study shows that caveolae contribute to GBM invasiveness in response to osmotic and hydrostatic pressure. Both types of pressure are a feature of GBM and likely to elicit both common and distinct activation pathways. This is compatible with the known functions of caveolae, which buffer membrane tensions,^22^ regulate mechanical stress‐induced signalling and the actin cytoskeleton,^46^ and may shelter membrane receptors.^47^ Together with our previous report that caveola‐forming proteins regulate matrix‐degrading enzymes in GBM in the absence of mechanical stress, our current results point to caveolae as a potential new target in the treatment of GBM.

## CONFLICT OF INTEREST

The authors confirm that there are no conflicts of interest.

## AUTHOR CONTRIBUTION

WP, RGP and MOP conceptualized and designed the study. WP, JQ, ZDN, KM and CF carried out experimental work. WP, RGP, JMH and MOP analysed and interpreted the data. WP, PNS, GJR, RGP, KM, JMH and MOP wrote the article and critically revised the article.

## Data Availability

The data that support the findings of this study are available from the corresponding author upon reasonable request.
