# Supplementary figures and images for: A role for caveola‐forming proteins caveolin‐1 and CAVIN1 in the pro‐invasive response of glioblastoma to osmotic and hydrostatic pressure
Source: J Cell Mol Med. 2020 Feb 17;24(6):3724–38. doi: 10.1111/jcmm.15076 (PMC7131935; doi:10.1111/jcmm.15076)

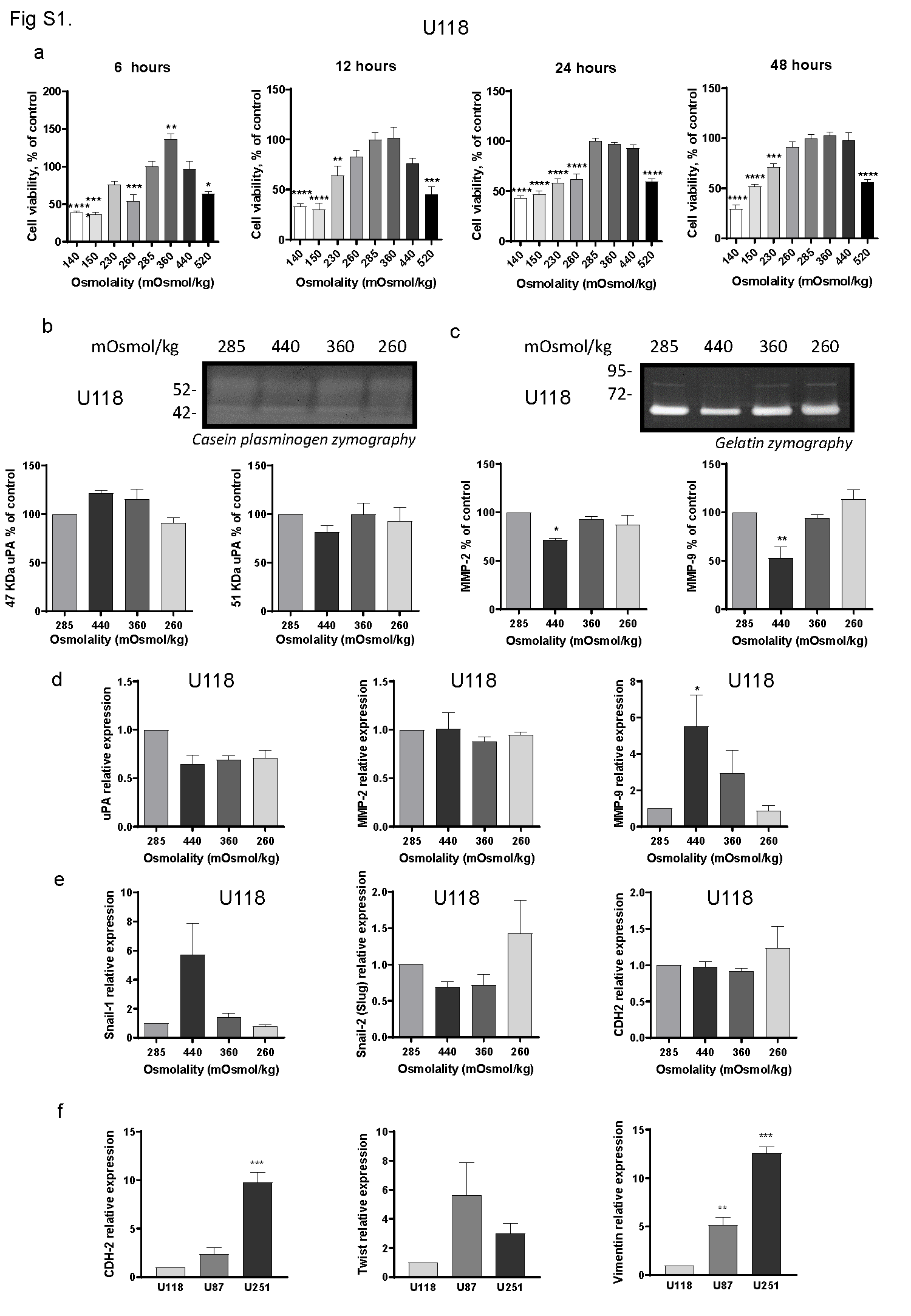

Supplement: Supplementary file 1 [file JCMM-24-3724-s001.tif]

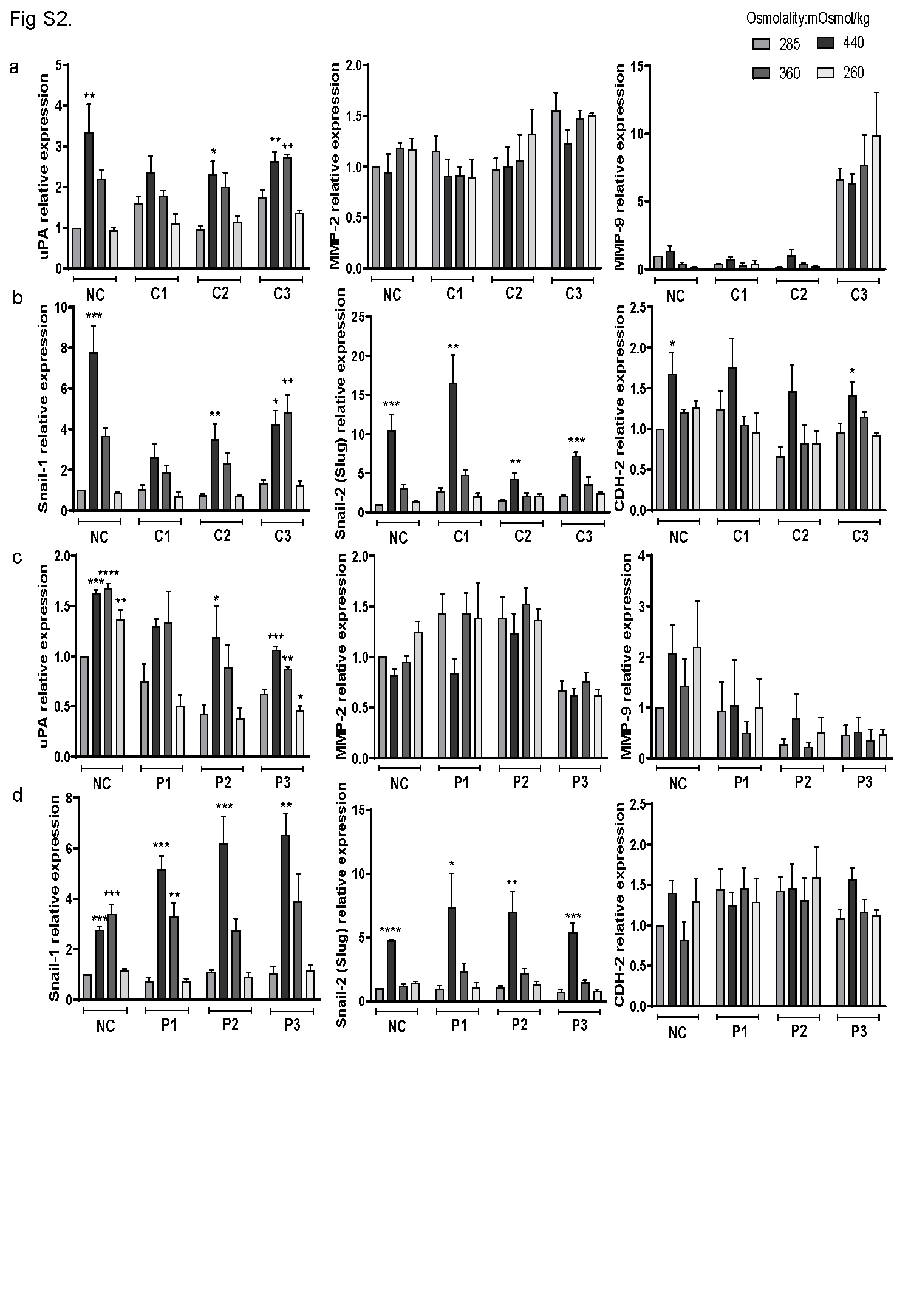

Supplement: Supplementary file 2 [file JCMM-24-3724-s002.tif]

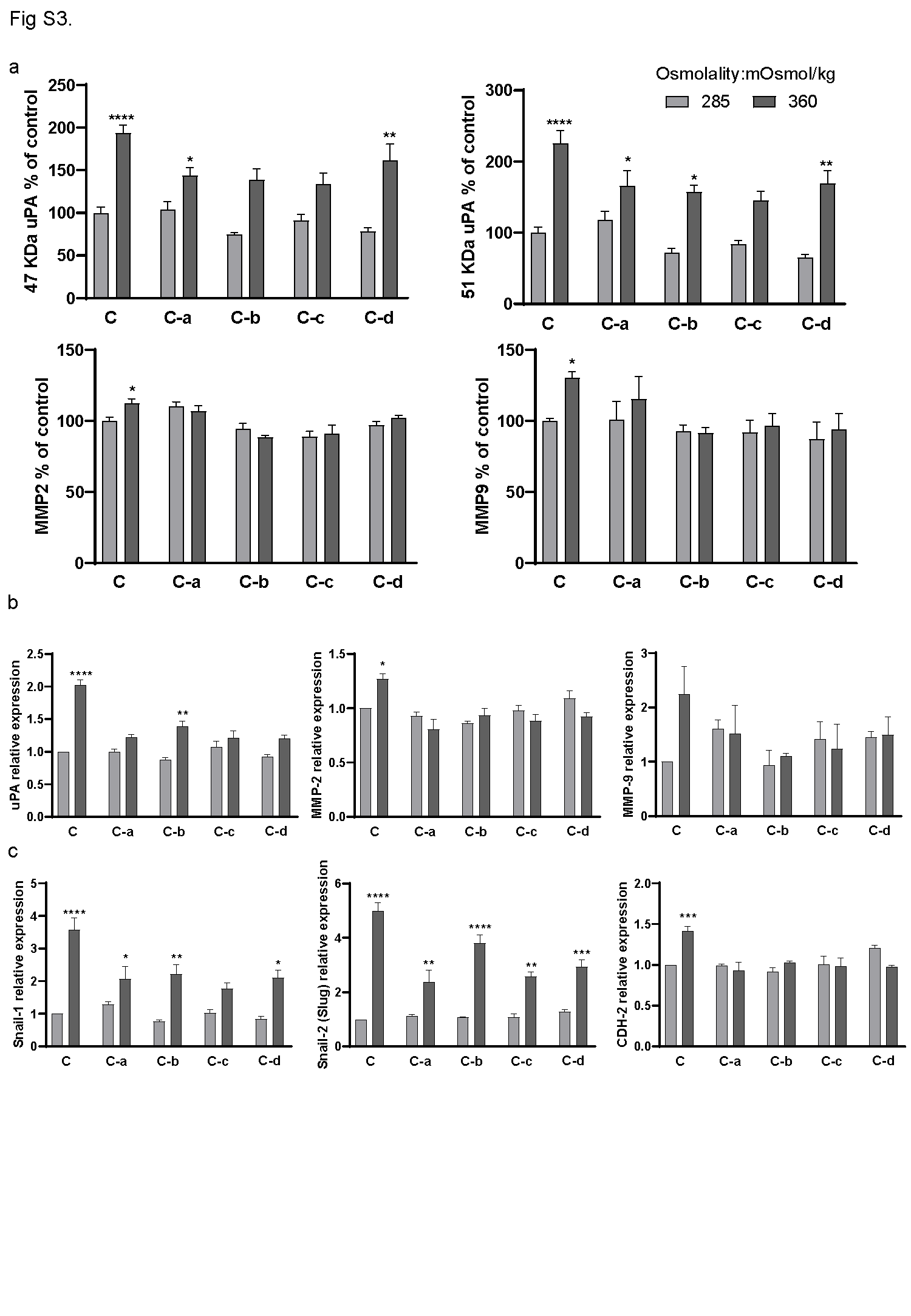

Supplement: Supplementary file 3 [file JCMM-24-3724-s003.tif]

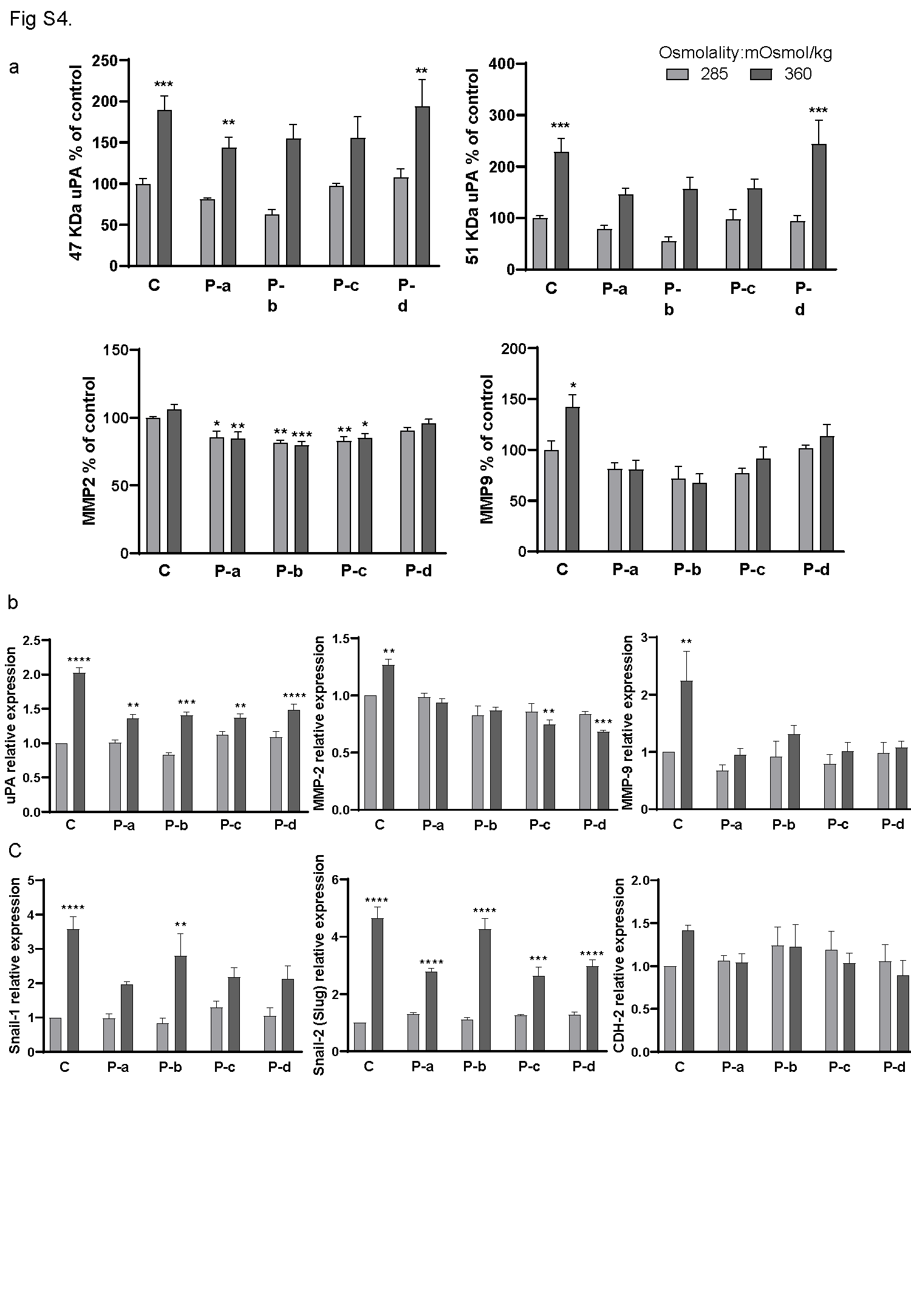

Supplement: Supplementary file 4 [file JCMM-24-3724-s004.tif]

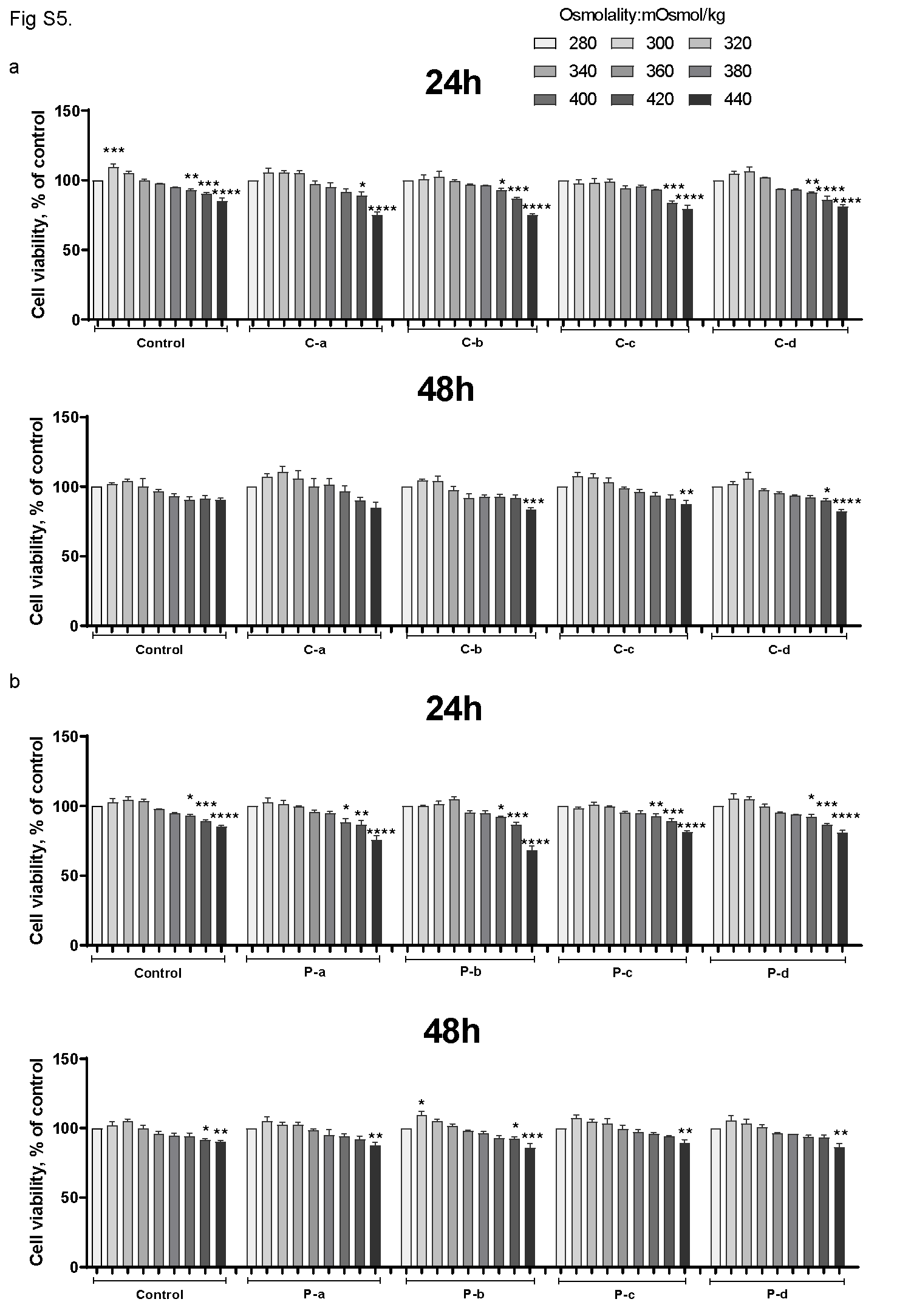

Supplement: Supplementary file 5 [file JCMM-24-3724-s005.tif]

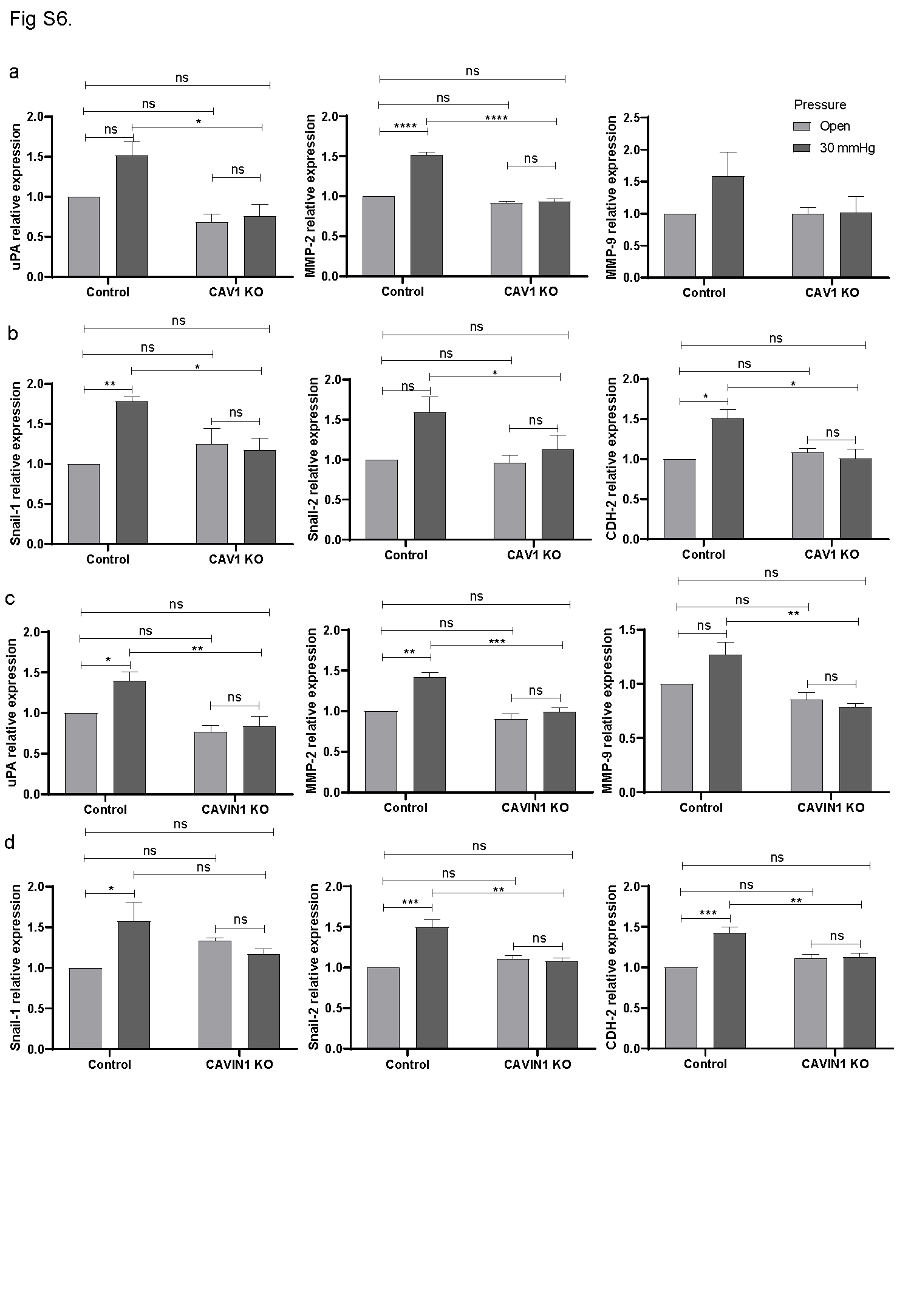

Supplement: Supplementary file 6 [file JCMM-24-3724-s006.tif]
